# Real-Time Reconstruction of HIFU Focal Temperature Field Based on Deep Learning

**DOI:** 10.34133/bmef.0037

**Published:** 2024-03-21

**Authors:** Shunyao Luan, Yongshuo Ji, Yumei Liu, Linling Zhu, Haoyu Zhou, Jun Ouyang, Xiaofei Yang, Hong Zhao, Benpeng Zhu

**Affiliations:** ^1^ School of Integrated Circuits, Laboratory for Optoelectronics, Huazhong University of Science and Technology, Wuhan, China.; ^2^ HIFU Center of Oncology Department, Huadong Hospital Affiliated to Fudan University, Shanghai, China.

## Abstract

*Objective and Impact Statement*: High-intensity focused ultrasound (HIFU) therapy is a promising noninvasive method that induces coagulative necrosis in diseased tissues through thermal and cavitation effects, while avoiding surrounding damage to surrounding normal tissues. *Introduction*: Accurate and real-time acquisition of the focal region temperature field during HIFU treatment marked enhances therapeutic efficacy, holding paramount scientific and practical value in clinical cancer therapy. *Methods*: In this paper, we initially designed and assembled an integrated HIFU system incorporating diagnostic, therapeutic, and temperature measurement functionalities to collect ultrasound echo signals and temperature variations during HIFU therapy. Furthermore, we introduced a novel multimodal teacher–student model approach, which utilizes the shared self-expressive coefficients and the deep canonical correlation analysis layer to aggregate each modality data, then through knowledge distillation strategies, transfers the knowledge from the teacher model to the student model. *Results*: By investigating the relationship between the phantoms, in vitro, and in vivo ultrasound echo signals and temperatures, we successfully achieved real-time reconstruction of the HIFU focal 2D temperature field region with a maximum temperature error of less than 2.5 °C. *Conclusion*: Our method effectively monitored the distribution of the HIFU temperature field in real time, providing scientifically precise predictive schemes for HIFU therapy, laying a theoretical foundation for subsequent personalized treatment dose planning, and providing efficient guidance for noninvasive, nonionizing cancer treatment.

## Introduction

High-intensity focused ultrasound (HIFU) is a noninvasive technique for tumor ablation [1]. HIFU tumor therapy represents an advanced interdisciplinary fusion of physical acoustics and medicine [2]. Its fundamental principle leverages the strong penetration and precise directional properties of ultrasound (US) waves to concentrate US energy within a specific focal region inside the body, typically at the millimeter scale [3]. This energy concentration induces thermal and cavitation effects, leading to a rapid increase in temperature within the focal region, resulting in coagulative necrosis. Ultimately, the objective of ablation is accomplished through mechanical dissolution, absorption, or fibrosis [4]. HIFU stands out for its nonradiative, noninvasive nature and real-time capabilities [1]. In the context of tumor treatment, it helps prevent organ adhesions, infections, and bleeding, thereby reducing the risk of tumor cell metastasis. This, in turn, alleviates patient suffering and improves their overall quality of life.

During the actual HIFU treatment process, biological tissues absorb acoustic energy and convert it into thermal energy [1]. Measurement and control of tissue temperature within the focused US focal region are crucial for the effectiveness of HIFU treatment [5]. However, due to the complex nature of nonuniform biological media (including bone, muscle, fat, and blood vessels), which influence sound propagation and exhibit differences in acoustic absorption, it is currently challenging to achieve accurate and real-time monitoring of the temperature distribution within the HIFU focal region [6,7]. As a result, it is difficult to plan effective US dosages (such as acoustic power and treatment duration) scientifically and precisely for patients. At present, the dosimetry planning for HIFU treatments relies more on the expertise of medical professionals. They typically set the basic treatment parameters based on the tumor’s location relative to the body surface and adjust acoustic parameters according to the patient’s symptoms or the risk factors associated with surrounding organs [8]. However, due to lack of “scientific precision”, HIFU treatments can sometimes result in incomplete target ablation or damage to surrounding normal tissues due to excessively high ablation temperatures [6–8]. This, to some extent, affects the efficacy of HIFU treatments. Therefore, the accurate and real-time monitoring of temperature within the HIFU focal region holds significant scientific importance and practical value for clinical cancer treatment.

Purely theoretical modeling methods usually employed finite element analysis and nonlinear transient thermal analysis to directly derive the thermal model and then obtain the temperature distribution within the focal region based on the energy parameters of the HIFU transducer [9]. However, theoretically derived methods ignore the complex effects of nonuniform biological media on sound propagation and absorption and are insufficient to accurately determine the temperature distribution within the tissue during HIFU therapy. Given that the absorption of acoustic energy within the focal region primarily manifests in the distribution of the thermal field (temperature field), a logical approach is to employ a thermocouple array for direct temperature measurements within the HIFU focal region [10,11]. Nevertheless, using thermocouples directly has several drawbacks. Firstly, this method is invasive, causing discomfort to patients and posing a risk of cancer cell metastasis, which contradicts the noninvasive concept of HIFU technology. Secondly, during HIFU treatment, there is a risk of thermocouple damage, and the presence of thermocouple probes can scatter US waves, ultimately affecting treatment effectiveness [12]. Most importantly, in clinical HIFU treatment, thermocouples may not always be accurately placed in the targeted tissue area [13]. Magnetic resonance imaging (MRI), as a noninvasive technique, is a feasible approach for monitoring temperature within the HIFU target area. Its principle involves applying radiofrequency pulses to the tissue within the focal region and determining tissue temperature by detecting the spin-lattice relaxation time of hydrogen protons [6,14]. This method offers a relatively high spatial resolution but also comes with certain challenges. Firstly, MRI has a low time resolution (0.3 fps) and frame rate (0.1 to 1 Hz), making real-time monitoring of HIFU treatment difficult [15]. Secondly, due to the inherent limitations of MRI, it is not suitable for treating patients with metal implants, pregnant individuals, and others. Furthermore, the high cost of treatment limits the widespread application of this method. In summary, whether it is through purely theoretical models, thermocouple temperature measurement methods, or MRI temperature monitoring techniques, achieving precise and real-time monitoring of the temperature distribution within the HIFU focal region remains challenging.

With the rapid development of artificial intelligence, deep learning–based medical image processing provides promising solutions in many data-driven clinical application challenges [16–18]. For example, big data technology enables breaking through the limits in various aspects such as tumor identification [19], super-resolution reconstruction [20], slice staining techniques [21], and treatment planning [22], to resolve the challenge mentioned above to monitor the temperature distribution in clinical applications. In this paper, a temperature distribution reconstruction strategy based on a deep multimodal teacher–student (MMTS) model is proposed to establish a nonlinear mapping relationship from ultrasonic echo signal to temperature. To fully validate the robustness of MMTS, we chose 3 phantoms, 3 in vitro porcine loin samples, and 2 in vivo Ba-Ma pigs to validate it. In summary, MMTS provides researchers with a novel strategy that enables fast and precise HIFU focal region temperature distribution reconstruction, thus effectively increasing reproducibility and scalability in HIFU thermotherapy research. Application areas include, but are not limited to, tumor ablation, cancer metastases, dose planning, drug delivery, particle implantations, and organ-at-risks studies.

## Results

### In phantoms temperature field reconstruction during HIFU treatment

**Fig. 1.**
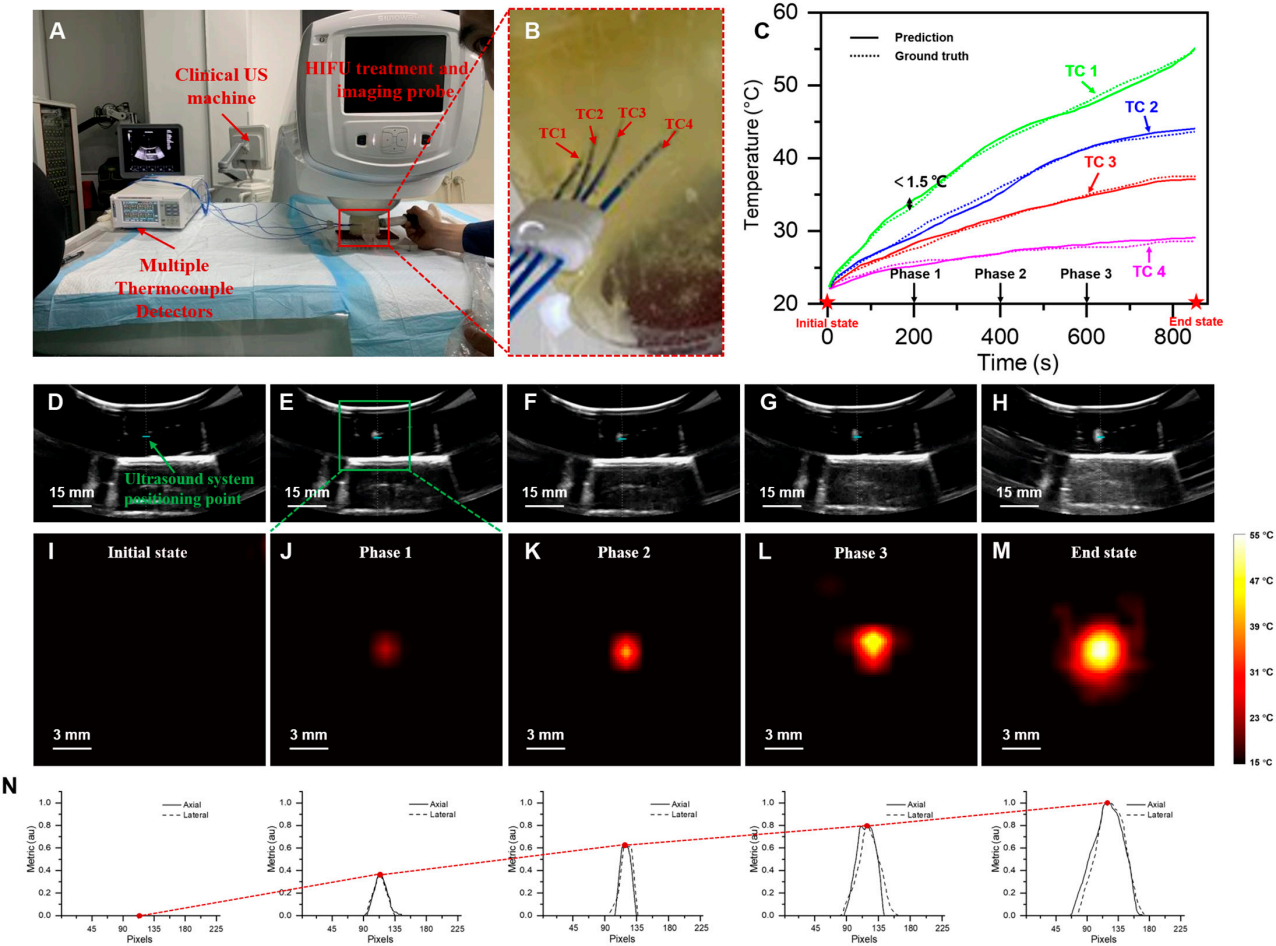
Real-time temperature field reconstruction of a phantom during HIFU treatment. (A) Experimental system. (B) Distribution of thermocouples in the phantom. TC1 to TC4 represent thermocouple numbers. (C) Comparison of MMTS-predicted temperatures with thermocouple-measured temperatures. (D to H) B-mode US images during HIFU treatment at different temporal nodes. (I to M) 2D temperature fields reconstructed by MMTS during HIFU treatment at different temporal nodes. (N) The interpolated profiles of axial and lateral at the focal region based on (I) to (M).

To assess the temperature field reconstruction capability, we constructed a HIFU diagnosis-treatment-temperature measurement integrated system, as shown in Fig. 1A. Initially, the handheld external US probe was employed to position the thermocouples, ensuring that all 4 thermocouple tips were in the same plane. Subsequently, the HIFU treatment and Imaging probe system was controlled to the correct position using a 3-axis electric platform. Finally, using an external trigger signal to records periodic temperature and echo signal (see Operation sequence for system). The distribution of thermocouples in the phantom is depicted in Fig. 1B. We utilized the thermocouples to record the temperature variations during the HIFU treatment (dashed line) and compared them with temperature results predicted by the MMTS strategy (solid line), as shown in Fig. 1C, with MMTS prediction errors being less than 1.5 °C. Based on Fig. 1C, 5 temporal nodes were selected to reconstruct the spatiotemporal temperature distribution within the HIFU focal region, as shown in Fig. 1I to M. With the increase of HIFU treatment time, the temperature field range expanded gradually, with the thermal scale map gradually intensifying. This trend is generally consistent with the region where vaporization occurs (white highlighting near the ultrasound system positioning point (USPP)] in the ultrasound B-mode imaging (UBI) (Fig. 1D to H). Figure 1N quantitatively demonstrates the interpolated profiles of axial and lateral at the focal region. The red dashed line reflects the variation trend of the highest temperature point in Fig. 1I to M, which generally corresponds to the heating process in Fig. 1C. Considering the tissue’s degeneration at the focal region during HIFU treatment, leading to altered heat transfer properties, the reconstructed temperature field deviates from the HIFU system’s preset focal size (3 mm × 3 mm × 5 mm).

### In vitro temperature field reconstruction during HIFU treatment

**Fig. 2.**
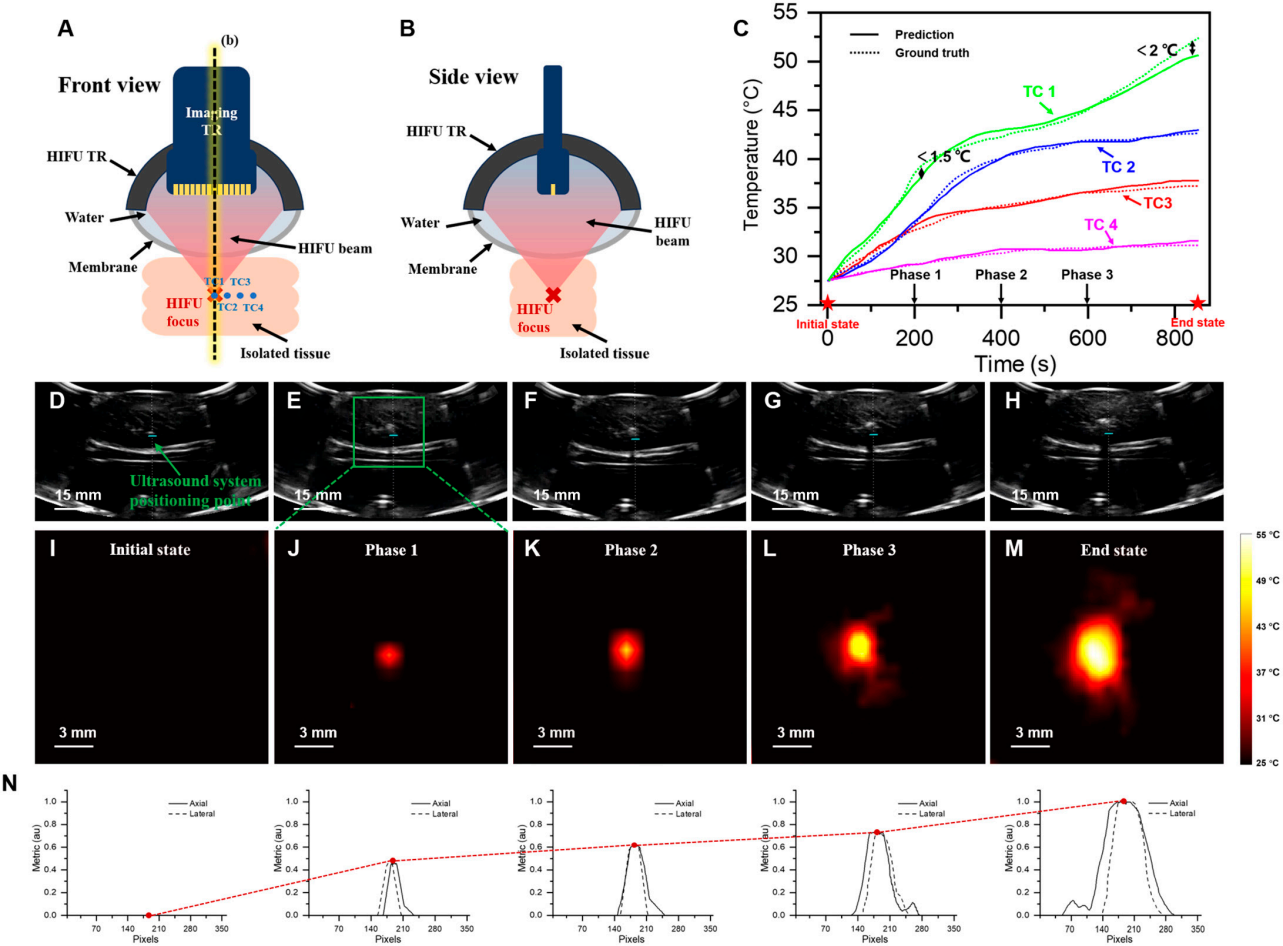
In vitro real-time temperature field reconstruction during HIFU treatment. (A) The front and (B) side views of the integrated transducer. (C) Comparison of MMTS-predicted temperatures with thermocouple-measured temperatures. (D to H) B-mode US images during HIFU treatment at different temporal nodes. (I to M) 2D temperature fields reconstructed by MMTS during HIFU treatment at different temporal nodes. (N) The interpolated profiles of axial and lateral at the focal region based on (I) to (M).

To assess the reconstruction ability of the temperature field in isolated tissues, similar to the phantoms experiment, four thermocouples were arranged parallelly and inserted into the fresh pork loin, following which therapeutic HIFU waves were emitted using an integrated transducer. The front and side views of the integrated transducer are shown in Fig. 2A and B, respectively. Figure 2C illustrates the temperature-time variations recorded by the 4 thermocouples and predicted by the MMTS. Within the 0- to 600-s timeframe, the deviation between predicted and actual temperatures remained below 1.5 °C. As temperatures continued to increase, the disparity between MMTS-predicted temperatures and actual temperatures gradually increased. At approximately 52 °C, significant tissue degeneration occurred internally, leading to more intricate echo signals, and reaching a maximum temperature disparity of 2 °C. Based on Figure 2C, 5 temporal nodes were selected to reconstruct the temperature distribution within the HIFU focal region, as illustrated in Fig. 2I to M. For better visualization, temperature values were normalized within the [0-1] range, with an initial temperature matrix set at zero. With increasing HIFU treatment time, the temperature field range expanded gradually, and the thermal scale map intensified gradually, the temperature field gradually assumed an elliptical shape, with the z-axis as the long axis. Considering the preset focal size of the HIFU system (3 mm × 3 mm × 5 mm), indicating a slightly longer temperature distribution along the z-axis than the x and y axes, this aligns with the results presented in Fig. 2I to M. Figure 2D to H shows the UBI of the initial state,200, 400, and 600 s, and the end state of the HIFU treatment. Within these images, the region of tissue vaporization is distinctly observable positioned at the upper-left region of the USPP. As the treatment duration progresses, the vaporization region gradually moves away from the USPP. This phenomenon is due to tissue displacement because of coagulative necrosis at the focal region. Reconstruction of a 2-dimensional (2D) temperature field solely based on amplitude information (UBI) would be disrupted by this situation. Conversely, the MMTS effectively integrates multimodal data, significantly mitigating distortions in the temperature field caused by tissue displacement, as illustrated in Fig. 2I to M. Figure 2N quantitatively presents the axial and lateral interpolated profiles at the focal region. The red dashed lines’ trends between the subfigures align consistently with the heating process in Fig. 2C).

### In vivo temperature field reconstruction during HIFU treatment

**Fig. 3.**
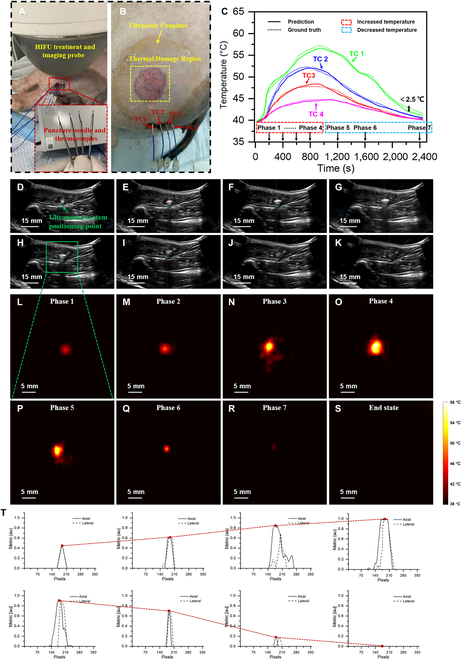
In vivo real-time temperature field reconstruction during HIFU treatment. (A) Schematic of in vivo experiment. (B) Thermal damage region and thermocouple insertion location. (C) Comparison of MMTS-predicted temperatures with thermocouple-measured temperatures. (D to H) B-mode US images during HIFU treatment at different temporal nodes. (I to M) 2D temperature fields reconstructed by MMTS during HIFU treatment at different temporal nodes. (N) The interpolated profiles of axial and lateral at the focal region based on (I) to (M).

For in vivo validation, we analyzed the temperature distribution within live tissues during the HIFU treatment process. Considering the influence of animal skin on thermocouple insertion into tissue layers, we employed a puncture needle along with a cannula for penetrate. Then, we positioned, adjusted, and confirmed the thermocouple position by an external US probe, as shown in Fig. 3A. Figure 3B demonstrates the HIFU thermal damage area. Figure 3C shows the temperature-time changes recorded by the 4 thermocouples and predicted by the MMTS. Distinguishing from the phantoms and in vitro experiments, our in vivo experiment involved a closed-loop HIFU treatment process, including both heating and cooling phase. Figure 3C shows that within 0 to 900 s, the HIFU treatment system continuously delivered therapeutic waves, resulting in a progressive temperature increase. During this phase, the difference between the predicted temperature and the true temperature at the positions of TC2, TC3, and TC4, which are farther away from the focal region, is less than 1 °C. The predicted error at TC1, significantly influenced by coagulative necrosis, exhibited a relatively larger deviation (2 °C). The period from 900 to 2,500 s denotes the cooling phase, predominantly achieved through heat conduction and animal metabolism. It was observed that the predicted errors at TC2, TC3, and TC4 locations were relatively minor, with an average error within 1 °C. However, due to tissue heterogeneity and hemodynamics, the error at TC1 was larger, averaging within 2.5 °C.

Based on Fig. 3C, we selected 8 temporal nodes to reconstruct the temperature distribution within the HIFU focal region, as depicted in Fig. 3L to S, where Fig. 3I to O represents the heating phase, and Fig. 3P to S represent the cooling phase. Please note that due to coordinate size limitations, Fig. 3S represents the end state, displaying the reconstructed temperature field at 3,000 s. For visual comprehension, we normalized temperatures within the [0-1], initializing both the initial- and end-state temperatures as zero matrices. At 0 to 900 s, the HIFU continuously delivered treatment waves, the reconstructed temperature field expanded gradually, the thermal scale map intensified gradually, and the temperature field distribution obeys the preset focal size of the HIFU system. From 1,200-s end state, the HIFU transducer ceased operation, the focal region temperature steadily decreased, resulting in the gradual reduction of the reconstructed temperature fields and a decrease in the thermal color scale. Figure 3D to G represents UBI during the heating phase, while Fig. 3P to S depict UBI during the cooling phase. Fig. 3T quantitatively presents the axial and lateral interpolated profiles at the focal region. The red dashed trends among the subfigures align consistently with the heating and cooling processes from Fig. 3C, further confirming the feasibility and accuracy of MMTS in reconstructing 2D temperature fields in animals.

### Computational complexity analysis

To demonstrate the real-time viability of the model, we conducted an analysis of the computational times of unimodal student knowledge distillation module (USKD) in phantoms, in vitro, and in vivo experiments. Table shows the time consumed to reconstruct a frame of temperature field by different numbers of USKDs. Our observations revealed that a 24GB 3090Ti workstation can concurrently run up to 480 USKDs. Comparatively, this parallel operation drastically reduces the computational time by 480 times when contrasted with using a single USKD, achieving a 0.204-s delay, effectively meeting clinical real-time standards. We contend that with sufficient computing resources, such as employing a 48GB workstation or an integrated workstation, the computational times can be further reduced, even to the microsecond level.

**Table.** The time consumed to reconstruct a frame of temperature field by different numbers of USKDs

Number of USKD	Phantoms	In vitro	In vivo
1	40.454 s	97.89 s	97.89 s
60	0.674 s	1.631 s	1.631 s
120	0.337 s	0.816 s	0.816 s
480	0.084 s	0.204 s	0.204 s

## Discussion

In this study, we propose a deep MMTS model, a simple and lean deep learning-enabled architecture for reconstructing the focal temperature field in HIFU treatment. Temperature field reconstruction is not only a highly precise task but also inherently challenging, especially under noninvasive, real-time, and universally applicable conditions. Therefore, there is an urgent need for reliable methods to obtain high-quality HIFU temperature fields. Unlike commonly used methods such as thermocouples, MRI, and US-based temperature measurements, the MMTS employs an end-to-end lightweight deep neural network, which successfully avoids cancer cell metastasis caused by thermocouple invasion into tissues and US image interference caused by tissue vaporization during HIFU treatment, and has good generalizability (for metal implanted patients, pregnant women, and children, etc.) and real-time (0.1 to 0.2 s) performance. We evaluated the model performance in phantom, in vitro, and in vivo experiments, revealing that MMTS achieves accuracy on par with thermocouples. Crucially, it surpasses 3 key barriers: noninvasiveness, real-time, and universality. This method is not tied to specific imaging modalities and remains insensitive to parameters such as imaging resolution, highlighting its broad applicability.

HIFU therapy primarily involves 3 main aspects: (a) Inducing localized tissue heating resulting in coagulative necrosis of abnormal cells. (b) Control the temperature within the treatment area to ensure appropriate cooling. (c) Gradual return to normal temperature and initiation of healing after the treatment. Therefore, in our in vivo experiments, we reconstructed the temperature field during the heating and cooling phases of HIFU, selecting 4 points to compare their differences against thermocouple temperature measurements. We observed that during the HIFU treatment process, the coagulative necrosis in the lesion area forces the tissue displacement, particularly evident during the cooling phase. Sole reliance on radio frequency (RF) data as the input data (a common practice in the HIFU temperature measurement region) could intricately affect temperature analysis. However, by choosing multimodal US echo signals as the input data, MMTS effectively circumvented temperature field distortion caused by tissue displacement. While algorithms constructed based on RF data might helpful for model development, it is only through referencing multimodal data that efficient generalization performance can be truly achieved.

Future work may explore avenues to further enhance robustness and generality for achieving 3D temperature field reconstruction. Potential approaches include leveraging multiple, more diverse datasets (e.g., collected across different laboratories using varying modalities), 3D convolutions (which may assist in handling tissue spatial structures but require more training data for convergence), US matrix probes (to acquire 3D US echo signals), and collaborate MRI (requires magnetic field shielding). The MMTS, as a lightweight deep neural network, will aid in the real-time and accurate reconstruction of the HIFU 2D temperature field, facilitating advancements in many clinical applications such as tumor ablation, dose planning, safety monitoring, and efficacy assessment. It has low barriers to adoption, has low computational requirements, and necessitates no manual intervention or parameter tuning. In summary, as a tool, MMTS and the findings of this study make significant contributions to enhancing the scalability of biomedical research, reducing biases, and promoting noninvasive cancer therapies.

## Materials and Methods

### System design

**Fig. 4.**
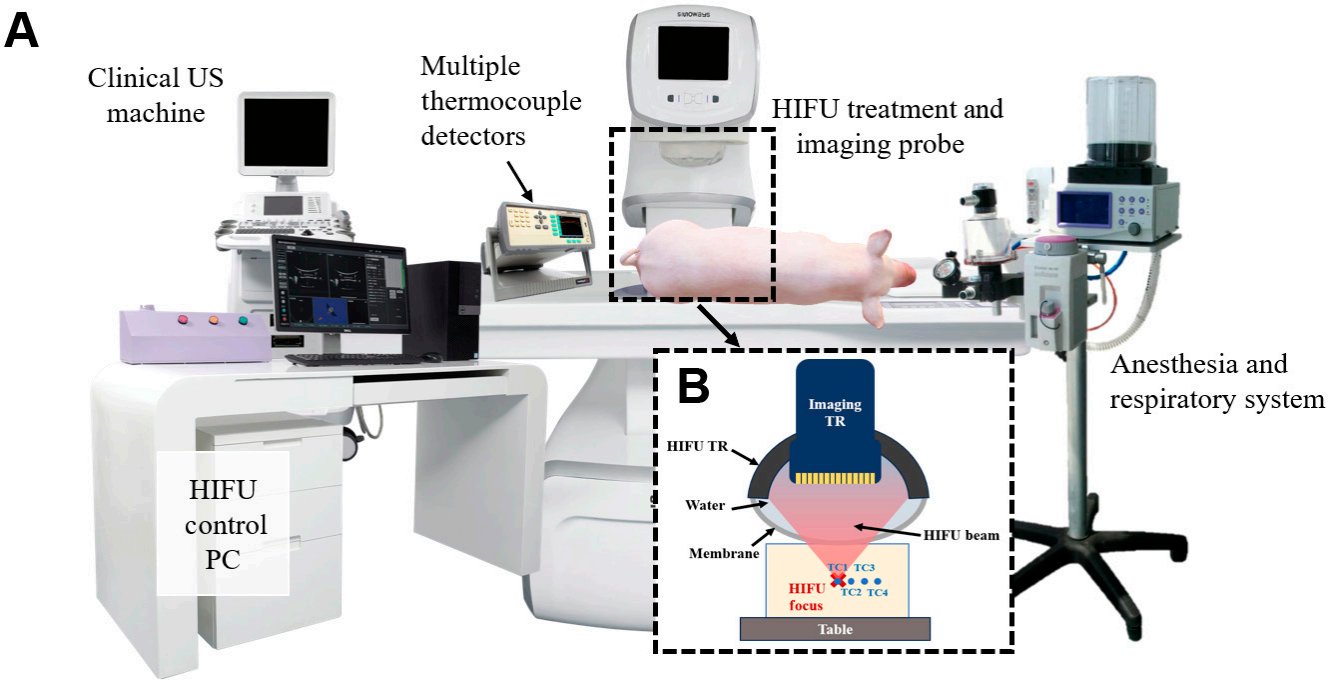
Real-time MTD and US imaging system synchronized with HIFU treatment machine. (A) System architecture. (B) The HIFU treatment transducer and US imaging transducer coaxially integrated into a single probe.

To acquire real-time temperature data and ultrasonic echo signals during HIFU treatment, we integrated and synchronized an US diagnostic system, a temperature detection system, and the HIFU treatment system, as depicted in Fig. 4. The temperature detection and US diagnostic systems consist of a sampling-rate-tunable multiple thermocouple detectors (MTD) (TCP-16XL, Spectra Instruments Co., Ltd., China) and a clinically US machine (MyLab ClassC, Esaote, Italy), as per the National Medical Products Administration registration number 20163061992. The HIFU treatment system is equipped with 2 types of US transducers: a 128-element linear array transducer (Composite L12-4 38, SINOWAYS, China) utilized for acquiring US echo signal, and an 6-element focused transducer (PA230E, SINOWAYS, China) employed for generating therapeutic HIFU waves. A computer (610L, Advantech, China) controls the focused transducer to produce therapeutic HIFU waves with a central frequency of 1 MHz, sound intensity of 5 kW/cm^2^, and a duty cycle of 65%. The linear array transducer is interfaced with the US diagnostic system for image reconstruction and to acquire US echo signals. Both transducers are coaxially integrated coaxially integrated into a single probe, the positioning of the probe is controlled by a 3-axis electric platform, and the probe is sealed with an acoustically transparent membrane filled with deionized water to achieve acoustic coupling, as depicted in Fig. 4B. During in vivo experiments, an isoflurane continuous anesthesia for the animals is generated using a respiratory anesthesia machine (GSM-IIA, Hongrunda, China). Throughout the entire experimental procedure, a medical US coupling agent (Shanghai Shenfeng Medical Healthcare Products Co., Ltd.) is utilized to match the acoustic impedance between the membrane and the target area.

### Operation sequence for system

**Fig. 5.**
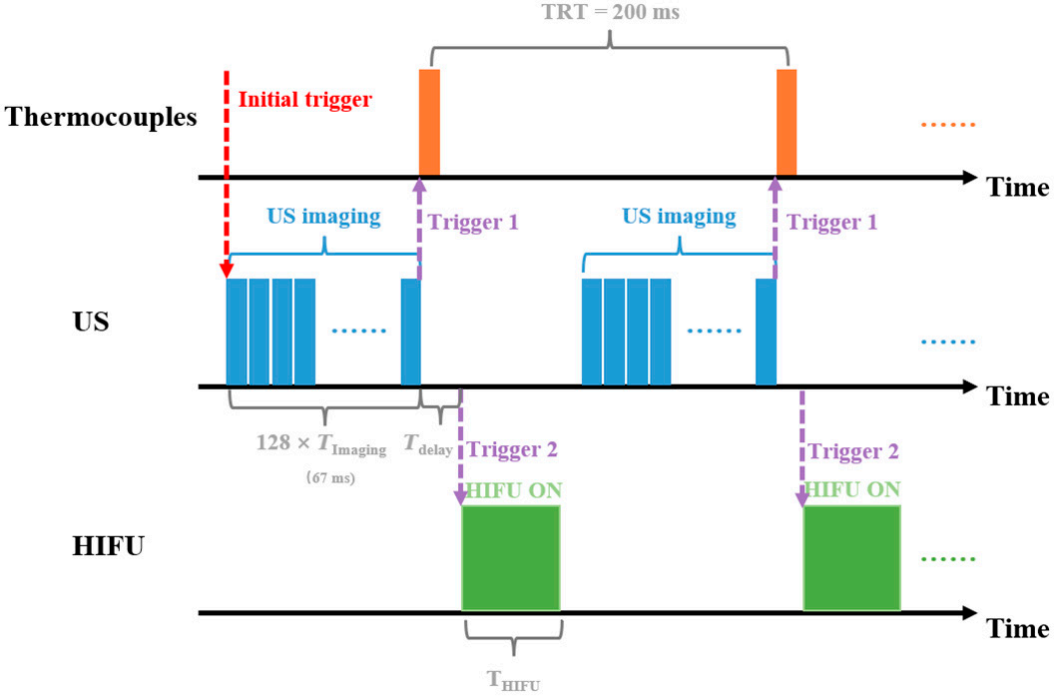
Operation sequence for acquiring temperature data and US echo signals during HIFU treatment. *T*_Imaging_: Signal reception time for single-element. *T*_Dely_: The time delay between the end of US imaging and the initiation of HIFU treatment. *T*_HIFU_: The time for HIFU treatment.

We have modified the operational sequence of the system, as shown in Fig. 5. By developing a MATLAB program, an artificial trigger signal (referred to as the “Initial Trigger” in Fig. 5) is sent to the US diagnostic system, then the US diagnostic system sequentially performs the following operations: (a) transmits and receives 128 US scan lines; (b) generates a trigger signal (labeled as “Trigger 1” in Fig. 5) and send it to the temperature detection system to record the target temperature; (c) waits for 3 ms to mitigate acoustic interference between the US echo wave and the HIFU treatment wave; (d) generates another trigger signal (termed “Trigger 2” in Fig. 5) and transmits it to the HIFU treatment system to produce the therapeutic HIFU wave. Each frame’s total imaging time is approximately 67 ms (comprising 128 data acquisitions for US imaging). The temperature repetitive time (TRT) is 200 ms. Considering the 67 ms for acquiring US echo signal and the 3-ms delay between the end of US imaging and the initiation of HIFU treatment, the remaining time for HIFU treatment (T_HIFU_) is 130 ms.

**Fig. 6.**
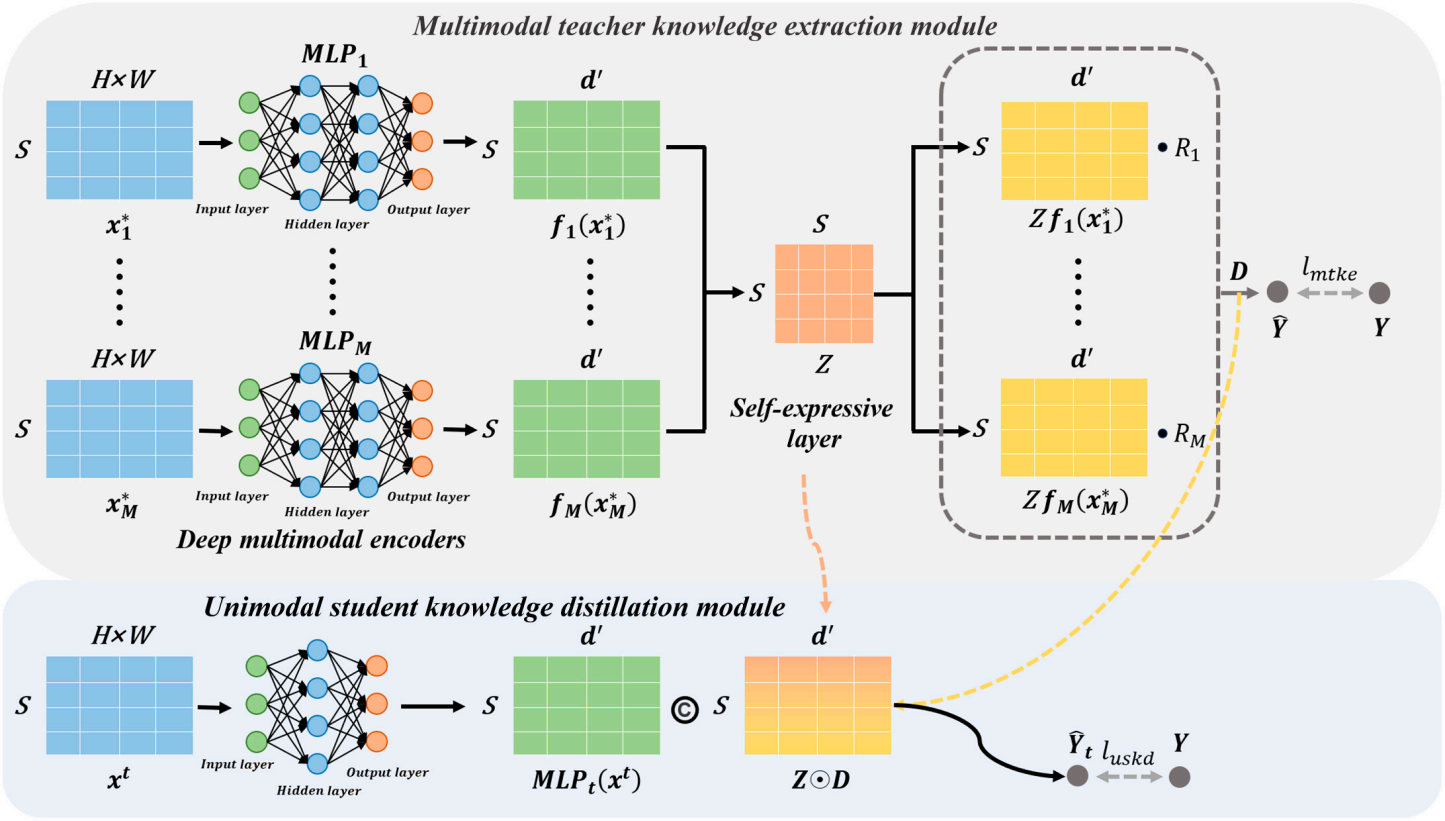
The framework of MMTS. In MTKE, the privileged modality data *X*^∗^ was mapped into $f_{M}\left(x_{M}^{*}\right)$ through the deep multimodal encoders. The self-expressive layer use coefficient matrix Z to analyze the intrinsic relationships among different samples. Then, the common space D is proposed to polymer the potential characterized in multimodal images. In USKD, the target modality data *x^t^*was mapped into *MLP_t_*(*x^t^*) by a 3-layer network. Then, we use the self-expressive coefficient matrix Z and the common space D to assist *MLP_t_*(*x^t^*) training. Finally, the USKD can decouple the association between *x^t^* and HIFU focal region temperature.

### Datasets

We conducted experiments employing 3 phantoms, 3 in vitro porcine loin samples, and 2 in vivo Ba-Ma pigs. The data from 2 phantoms, 2 in vitro samples, and one in vivo Ba-Ma pig were combined and divided into training and validation sets according to 7:3, the remaining data were used as the testing set.

#### Phantoms

The phantoms were prepared using a solution of (H_2_C=CHCONH)_2_CH^2^, (NH_4_)_2_S_2_O_8_, C_6_H_16_N_2_, and deoxygenated water. Additionally, 20 g of egg white was added to simulate tissue microstructures. Four thermocouples (perpendicular to the heating beam) were utilized to record the spatiotemporal temperature distribution. The thermocouples were connected to the temperature detection system, as depicted in Fig. 1A. The thermocouples, labeled TC1, TC2, TC3, and TC4, were gradually moved away from the heating area, with TC1 located at the focal spot, and the distance between adjacent thermocouples set at 3 mm. The linear US transducer (Composite L12-4 38) was positioned above the focal spot of the HIFU transducer.

#### In vitro

Fresh pork loin purchased from a local butcher shop was utilized to prepare experimental samples. US guidance (external probe) was employed to locate the region of flat, substantial tissue depth, and minimal fat content, designating it as the experimental area. The tissue samples were deoxygenated before inclusion into the experimental setup. Under US guidance, 4 thermocouples were inserted at equal distances (spaced 3 mm apart), equal depths, and parallelly into the interior of the pork tissue, reaching depths of 5 to 6 cm. Subsequently, an external US probe was used to reposition, adjust, and confirm that the tips of the 4 temperatures were situated on the same plane.

#### In vivo

In vivo experiments were conducted on two 25-kg female Ba-Ma pigs (Jiagan Biotech Co., Ltd., Shanghai, China) that were fasted for 12 h before the experiment. During the experiment, 3 ml of xylazine was injected into the muscle of the Ba-Ma pigs for anesthesia induction, using 3% isoflurane in oxygen maintaining anesthesia. US guided localization of the left gluteus maximus muscle of the Ba-Ma pigs, selected relatively flat, substantial tissue depth, and devoid of bony structures as the experiment’s region. Four needle insertion points aligned on the same plane were identified and marked on the skin surface using a marking pen. Using the tip of a surgical blade, incisions approximately 0.3 cm in length were made at each marked point. Under US guidance, a 15-G puncture needle with its cannula at the marked points was employed and penetrated to a depth of 5 cm; then the puncture needle was withdrawn, and the thermocouple was inserted through the cannula, as shown in Fig. 3A. All animal experiments were performed in compliance with the protocol (202310034S) approved by the Animal Welfare and Ethics Group, Department of Laboratory Animal Science, Fudan University.

### Training and inference details

The raw ultrasound data (RUD) encapsulates a diverse array of tissue information, encompassing echo time, echo amplitude, echo frequency, echo phase, and scattering characteristics. Simultaneously, it introduces a myriad of noise, spanning electronic noise, environmental noise, scattering noise, and motion noise [23]. Directly employing the RUD as input for neural networks may lead to network instability due to the influence of noise [24]. To optimize data efficacy and establish a robust connection with clinical applications, we extract echo amplitude, echo phase, and echo frequency, to generate UBI [25], ultrasound elastography imaging (UEI) [26], and ultrasound doppler imaging (UDI) [27]. In this paper, we call UBI, UEI, and UDI as privileged modalities data and call UBI as target modality data.

During the training process, we utilize the coordinates of the thermocouple as central points to extract 16 × 16 2D patches in privileged modalities data, and the temperatures as labels. During the inference process, we employ sliding window techniques on the target modality data to extract a 16 × 16 2D patch with a 90% overlap ratio. Please note that the 3-layer Multi-Layer Perceptron (MLP) architecture employed in the MMTS strategy, detailed in A deep multimodal teacher–student model, is highly suitable for parallel computation, and extremely fast inference, which enables real-time HIFU focal region temperature distribution reconstruction with sufficient computational resources.

## A deep multimodal teacher–student model

The proposed MMTS as shown in Fig. 6 comprises 2 modules: the Multimodal Teacher Knowledge Extraction Module (MTKE) and the USKD). The MTKE reconstructs multimodal images into a structured coordinated representation, consisting of 3 components: deep multimodal encoders, a self-expressive layer, and a deep canonical correlation analysis (DCCA) layer. The USKD is supported by the MTKE through the knowledge distillation scheme for effective learning. During the inference phase, the USKD can make accurate predictions without the requirement of multimodal images.

### Multimodal teacher knowledge extraction module

1. Deep Multimodal Encoders

Within the focal region, an increase in tissue temperature induces changes in acoustic parameters (such as echo velocity, echo frequency, and echo amplitude) [28]. The MLP, serving as a universal approximator, possesses the capability to approximate any continuous function, thereby enabling the learning and representation of intricate nonlinear relationships [29]. It is noteworthy that the robust nature of MLP allows it to simulate brain-like effects on an infinitely large scale [29]. This exceptional approximation and learning capability positions MLP as a highly promising tool for real-time temperature monitoring within the HIFU focal region. In this study, MLPs are employed as deep encoders to reconstruct multimodal images into a hierarchical representation.

Specifically, we define $X^{*}=\left\{x_{1}^{*},,,\ldots ,,,x_{M}^{*}\right\}\in R^{M\times S\times H\times W}$ as privileged modality data [30], where M, S, H, and W represent the number of modalities, samples, 2D patches height, and 2D patches width, and the label was defined as *Y* ∈ *R^S^*. Considering the heterogeneity of multimodal images, we have designed MLP module with the same architecture but varying parameters for different modalities. Specifically, *MLP_m_* corresponds to the $x_{m}^{*}\in R^{S\times H\times W},m=1:M$ modalities, which is a 4-layer network, with the input layer having *H* × *W* neurons, the hidden layer having *c* × *H* × *W* neurons, and the output layer having *d*^′^ neurons. Nonlinearly mapping *X*^∗^ to a hierarchical representation is achieved according to Eq. 1.

$$h_{m}^{i+1}=g\left(W_{m}^{i}h_{m}^{i}+b_{m}^{i}\right)$$ (1)

Where *i* = 1, 2, 3, $h_{m}^{1}=x_{m}^{*}$, *g* is the activation function, $W_{m}^{i}$ represents weights matrix, and the $b_{m}^{i}$ is biases vector. The final output is defined as $f_{m}\left(x_{m}^{*}\right)\in R^{S\times d\prime }$. The weights and biases *θ_m_* = {*W_m_*, *b_m_*} are updated by back propagation.

2. Self-expressive layer

When evaluating the thermal response of tissues, it is essential to consider a combination of factors, encompassing viscous losses, thermal conduction, and various forms of molecular relaxation processes [31]. These behaviors are all influenced by both the excitation time and the excitation frequency of the HIFU transducer [31]. Given the utilization of a single-frequency excitation at 1 MHz in this study, the excitation time emerges as a crucial independent variable affecting heat deposition in the focal region. Consequently, analyzing the intrinsic relationships among different samples (different excitation times) becomes particularly significant. The fundamental concept of the self-expressive layer is that each sample can be represented by a linear combination of others within the same subspace [32], which implies the self-expressive layer enables revealing the underlying relationships among the samples (excitation times).

To be specific, a self-expressive coefficient matrix *Z* ∈ *R*^*S*×*S*^ was defined to capture the potential relationships of the samples within the modalities and between the modalities. Then we combine the $f_{m}\left(x_{m}^{*}\right)$ with the self-expressive coefficient matrix as ${\mathrm{Zf}}_{m}\left(x_{m}^{*}\right)$, according to [33], can be obtained as Eq. 2.To avoid the trivial solution *Z* = *E*, where *E* is the identity matrix, we introduce a constraint *diag*(*Z*) = 0.

$$\begin{array}{l} {\mathrm{Zf}}_{m}\left(x_{m}^{*}\right)=f_{m}\left(x_{m}^{*}\right)\\ s.t.\mathrm{diag}\left(Z\right)=0 \end{array}$$ (2)

The total loss function of the self-expressive layer as shown in Eq. 3.

$$l_{s}=\lambda_{1}{\left\|Z\right\|}_{F}^{2}+\lambda_{2}\sum_{m=1}^{M}{\left\|{\mathrm{Zf}}_{m}\left(x_{m}^{*}\right)-f_{m}\left(x_{m}^{*}\right)\right\|}_{F}^{2}$$ (3)

Where *λ*_1_ and *λ*_2_ are the trade-off parameters. ${\left\|\cdot \right\|}_{F}^{2}$ are the matrix Frobenius norm. In particular, the first term serves to prevent overfitting, whereas the second term directs *Z* to have a self-expressive capability.

3. DCCA Layer

The raw US echo signal encapsulates contains various tissue information, including echo time, echo amplitude, echo frequency, echo phase, and scattering characteristics. Different tissue information corresponds to different modalities, representing distinct physical features, which are crucial for focal region temperature measurement [34]. To generate structural common spaces for multimodal images and polymer the potential characterized in multimodal images, we have introduced a DCCA layer. The objective function of DCCA as shown in Eq. 4.

$$\begin{array}{l} \min_{\left(D,,,Z,,,\theta ,,,P\right)}\sum_{m=1}^{M}{\left\|D-{\mathrm{Zf}}_{m}\left(x_{m}^{*}\right)R_{m}\right\|}_{F}^{2}\\ s.t.D^{T}D=I \end{array}$$ (4)

Where $D=\frac{\sum_{m=1}^{M}{\mathrm{Zf}}_{m}\left(x_{m}^{*}\right)R_{m}}{M}$ is the structural common space template, *R_m_* are reconstruct matrix. The details of *R_m_*will be mentioned later. Next, we reason about the final DCCA loss function based on Eq. 4.

We define the *m-th* modal output as $O_{m}={\mathrm{Zf}}_{m}\left(x_{m}^{*}\right)\in R^{S\times d\prime }$. According to statistical analysis, the $O_{m}^{T}O_{m}\in R^{d\times \prime d\prime }$ is called scatter matrix, and call $O_{m}{\left(O_{m}^{T}O_{m}\right)}^{-1}O_{m}^{T}\in R^{S\times S}$ the projection matrix. Then, the *r_m_I* ∈ *R*^*d* '  × *d* '^ was added to the projection matrix, as shown in Eq. 5.

$$\begin{array}{l} P_{m}=O_{m}{\left(O_{m}^{T}O_{m}+r_{m}I\right)}^{-1}O_{m}^{T}\\ \;=U_{m}\Sigma_{m}^{T}{\left(\Sigma_{m}\Sigma_{m}^{T}+r_{m}I\right)}^{-1}\Sigma_{m}U_{m}^{T} \end{array}$$ (5)

Where *P_m_* is projection matrix. $O_{m}\overset{\mathrm{SVD}}{\to }U_{m}\Sigma_{m}V_{m}^{T}$ is apply rank-r SVD [35] on *O_m_*, *U_m_* ∈ *R*^*S*×*r*^ and *V_m_* ∈ *R*^*r*×*d*'^ are the left and the right singular matrix, respectively. Σ*_m_* ∈ *R*^*r*×*r*^ is the singular diagonal matrix, which recorded r-largest feature values in *O_m_*. Then, the sum of *P_m_* can be defined as Eq. 6.

$$\begin{array}{l} P=\sum_{m=1}^{M}P_{m}\\ s.t.P^{T}P=I \end{array}$$ (6)

Where *P* contains the intrinsic connection of multimodal RF signals, *D* mentioned above as the structural common space template. Therefore, *D* necessarily encapsulates valuable information regarding the structural characteristics of *P*. Inspired by [36], we define *D* represents the left singular matrix of rank-r SVD on *P*, as shown in Eq. 7.

$$P\overset{\mathrm{SVD}}{\to }D\Sigma S^{T}$$ (7)

Where Σ ∈ R^*r*×*r*^ is the singular diagonal matrix contains *r* singular values {*s*_1_, *s*_2_…, *s_r_*}. *D* and *S* are the left and right singular matrices of Σ. When considering $R_{m}={\left(O_{m}^{T}O_{m}+r_{m}I\right)}^{-1}O_{m}^{T}D$, the objective function of DCCA in Eq. 4 may further be shown as Eq. 8.

$$\begin{array}{c} \begin{array}{l} \min_{\left(D,,,Z,,,\theta ,,,P\right)}\sum_{m=1}^{M}{\left\|D-{\mathrm{Zf}}_{m}\left(x_{m}^{*}\right)R_{m}\right\|}_{F}^{2}\\ \to \min\sum_{m=1}^{M}{\left\|D-O_{m}{\left(O_{m}^{T}O_{m}+r_{m}I\right)}^{-1}O_{m}^{T}D\right\|}_{F}^{2}\\ \to \min\left[\mathrm{mr}-\mathrm{Tr}\left(D^{T}\mathrm{PD}\right)\right]\\ \to \max\left(\mathrm{Tr}\left(D^{T}\mathrm{PD}\right)\right)\equiv \max\sum_{i=1}^{r}s_{i} \end{array} \end{array}$$ (8)

The loss function of DCCA can be shown as Eq. 9.

$$l_{d}=-\;\sum_{i=1}^{r}s_{i}$$ (9)

4. The Total Loss of Multimodal Teacher Knowledge Extraction Module

In the end, we use a projection mapping strategy to map *D* to $\hat{Y}\in R^{S}$, and use the MSE loss function [37,38] for temperature fitting. Therefore, the total loss function of MTKE as shown in Eq. 10, where *λ*_1_, *λ*_2_, *λ*_3_, and *λ*_4_ act as trade-off parameters, set as 1.0, 1.0, 1.0, 1.0.

$$l_{\text{mtke}}=\lambda_{1}{\left\|Z\right\|}_{F}^{2}+\lambda_{2}\sum_{m=1}^{M}{\left\|{\mathrm{Zf}}_{m}\left(x_{m}^{*}\right)-f_{m}\left(x_{m}^{*}\right)\right\|}_{F}^{2}-\lambda_{3}\sum_{i=1}^{r}s_{i}+\lambda_{4}\sum_{i=1}^{S}\frac{{\left(y-\hat{y}\right)}^{2}}{S}$$ (10)

5. Implementation details

The neural network using PyTorch was implemented, and experiments were performed on a small NVIDIA RTX3090Ti workstation equipped with 24GB of RAM. All model was trained by Adam with max iteration 10, 000, initial learning rate 1𝑒^−3^, and decayed exponentially with power 0.99. The batch size and the epochs were 16 and 80.

### Unimodal student knowledge distillation module

Utilizing a multimodal approach typically necessitates the use of an entire set of required modalities during testing to maintain performance, which can easily fail. However, in HIFU temperature field detection, obtaining a comprehensive set of high-quality multimodal data is often challenging due to the imperative consideration of real-time factors [33]. Consequently, leveraging a multimodal teacher model to assist a unimodal student model becomes particularly crucial in achieving accurate and real-time HIFU temperature predictions.

In this paper, we designed a USKD module, make full use of multimodal data to explore temperature prediction based on unimodal data. Specifically, we define *x^t^* ∈ *R*^*S*×*H*×*W*^ as target modality data, where S, H, and W represent the number of samples, 2D patches height, and 2D patches width. Please note that *x^t^* can be any modality in *X*^∗^, and here *x^t^* is UBI. *MLP_t_*(*x^t^*) corresponds to the *x^t^*, which is a 3-layer network, with the input layer, the hidden layer, and the output layer, having *H* × *W*, *c* × *H* × *W*, and *d*^′^ neurons, respectively. The output is defined as *MLP_t_*(*x^t^*) ∈ *R*^*S*×*d*'^. From the MTKE, we know that the self-expressive coefficient matrix *Z* contains rich multimodal sample intrinsic relationships, and the structural common space template *D* polymerized potential features of multimodal samples. After the MTKE is trained, we fix the parameters of the *Z* and *D*, then aggregate them as prior knowledge with *MLP_t_*(*x^t^*), as shown in Eq. 11.

$$O_{t}=\text{Concat}\left({\mathrm{MLP}}_{t}\left(x_{t}\right),,,\text{mapping}\left(Z⊙D\right)\right)$$ (11)

Where *mapping* denotes a one-dimensional convolutional layer for dimensional matching. ⊙ is the Hadamard product. Finally, we use a projection mapping strategy to map *O_t_* to ${\hat{Y}}_{t}\in R^{S}$, and use the MSE loss function for temperature fitting. Therefore, the loss function of USKD as shown in Eq. 12.

$$l_{\text{uskd}}=\sum_{i=1}^{S}\frac{{\left(y-{\hat{y}}_{t}\right)}^{2}}{S}$$ (12)

USKD contains the potential relationship and feature learned from privileged modalities by MTKE, and the temperature of focal regions can be directly obtained to support temperature field reconstruction.

## Data Availability

The data that support the findings of this study are available upon reasonable request from the authors.
