# Supplementary material for: Real-Time Reconstruction of HIFU Focal Temperature Field Based on Deep Learning
Source: BME Front. 2024 Mar 21;5:0037. doi: 10.34133/bmef.0037 (PMC10956737; doi:10.34133/bmef.0037)
Supplement: Supplementary 1 — Tables S1 to S3 Movie S1 [file bmef.0037.f1.docx]

**Supplementary Table.1.** Compare the temperature maximum error, temperature mean error and time consume under different patches sizes in phantoms.

|  | temperature maximum error | temperature mean error | time consume | | | |
| --- | --- | --- | --- | --- | --- | --- |
|  |  |  | 1 USKD | 60 USKD | 120 USKD | 480 USKD |
| 8×8 | 2.95 | 2.13 | 7.88s | 0.131s | 0.066s | 0.016s |
| 16×16 | 1.29 | 0.74 | 40.454s | 0.674s | 0.337s | 0.084s |
| 32×32 | 1.14 | 0.69 | 221.13s | 3.685s | 1.843s | 0.461s |
| 64×64 | 2.11 | 1.77 | 1371.72s | 22.862s | 11.431s | 2.858s |

**Supplementary Table.2.** Compare the temperature maximum error, temperature mean error and time consume under different patches sizes in *vitro*.

|  | temperature maximum error | temperature mean error | time consume | | | |
| --- | --- | --- | --- | --- | --- | --- |
|  |  |  | 1 USKD | 60 USKD | 120 USKD | 480 USKD |
| 8×8 | 3.03 | 2.55 | 14.61s | 0.243s | 0.122s | 0.003s |
| 16×16 | 1.47 | 0.87 | 97.89s | 1.631s | 0.816s | 0.204s |
| 32×32 | 1.36 | 0.82 | 483.96s | 8.066s | 4.033s | 1.008s |
| 64×64 | 2.33 | 1.83 | 2549.61s | 42.49s | 21.25s | 5.312s |

**Supplementary Table.3.** Compare the temperature maximum error, temperature mean error and time consume under different patches sizes in *vivo*.

|  | temperature maximum error | temperature mean error | time consume | | | |
| --- | --- | --- | --- | --- | --- | --- |
|  |  |  | 1 USKD | 60 USKD | 120 USKD | 480 USKD |
| 8×8 | 5.11 | 3.49 | 14.61s | 0.243s | 0.122s | 0.003s |
| 16×16 | 2.36 | 1.76 | 97.89s | 1.631s | 0.816s | 0.204s |
| 32×32 | 3.11 | 1.58 | 483.96s | 8.066s | 4.033s | 1.008s |
| 64×64 | 3.72 | 2.61 | 2549.61s | 42.49s | 21.25s | 5.312s |

**Supplementary Video.1.** Real-time changes of both the US B-mode images and the reconstructed temperature fields under the HIFU treatment
